# Assessment of Job Satisfaction and Intention to Quit Job Among Pharmacists in Saudi Arabia

**DOI:** 10.3390/pharmacy13060163

**Published:** 2025-11-05

**Authors:** Ashwaq Alharthi, Maha Aleiban, Abdulrahman Alwhaibi, Moureq Alotaibi, Yousef Almutairi, Sultan Alghadeer

**Affiliations:** 1 Corporate of Pharmacy Services, King Saud University Medical City, Riyadh 11472, Saudi Arabia; aalharthi1@ksu.edu.sa (A.A.); maleiban@ksu.edu.sa (M.A.); ysalmutairi@ksu.edu.sa (Y.A.); 2 Department of Clinical Pharmacy, College of Pharmacy, King Saud University, Riyadh 11451, Saudi Arabia; salghadeer@ksu.edu.sa; 3 Department of Pharmacology and Toxicology, College of Pharmacy, King Saud University, Riyadh 12746, Saudi Arabia; mralotaibi@ksu.edu.sa

**Keywords:** pharmacists, job satisfaction, turnover intention, Saudi Arabia, professional motivation, workplace environment

## Abstract

Background/Objectives: Job satisfaction is an essential element for organizational functions. Working entities would not effectively operate without employee contentment. This study aimed to determine the level of job satisfaction among pharmacists and investigate its correlation with demographic variables and professional personal experience. Methods: A cross-sectional online survey targeting registered pharmacists in Saudi Arabia was conducted from September to November 2024 using an IRB-approved structured questionnaire adapted from validated instruments. Reliability and validity were confirmed (Cronbach’s α = 0.8), and a target sample of 380 was calculated to ensure representativeness. Data were analyzed using descriptive statistics, chi-squared tests, and univariate and multivariate logistic regression analyses utilizing SPSS v28, with significance set at *p* < 0.05. Results: A total of 330 pharmacists responded to the survey, representing 86.8% of the calculated sample size. Of those, 57% were male and 68.5% were staffing pharmacists. More than half of participants had professional experience of ≤5 years (57.3%), while 31.8% had 5 to 15 years of experience. Approximately 60% of participants worked in shift systems and reported dissatisfaction with their pay (70%) and lack of benefits (66.7%). Of all participants, only 26.4% confirmed satisfaction with their job and no intention to quit, while 23% clearly reported job dissatisfaction and an intention to quit; the rest of the participants were undecided (50.6%). Significant correlations were found between job satisfaction and variables such as education, current position, organization type, monthly income, and professional experience. Additionally, most of the items assessing professional personal experience such as working in a shift system, working as a team member, gaining financial benefits, and having accomplishments or growth opportunities at work were significantly correlated with job satisfaction. Opportunities for professional development, promotion, and a positive work environment were also frequently selected as factors contributing to job satisfaction (60.6%, 75.2% and 75.5%, respectively). Interestingly, motivation showed minimal impact on participants’ opinions regarding job satisfaction and decisions over whether to quit their jobs. Finally, occupation and age were found to significantly influence work environments, promotions, and opportunities, which consequently impact participants’ satisfaction towards their jobs. Conclusions: Our findings indicate that Saudi pharmacists experience low-to-moderate job dissatisfaction, with a significant percentage considering quitting form their jobs. Improving monetary rewards, recognition, and career advancement opportunities could improve job satisfaction and retention in this crucial workforce.

## 1. Introduction

Job satisfaction is generally described as a positive emotional response toward work, based on an evaluation of work experience. It is a core element in employee work performance, retention, and the overall quality of care in health systems. In Saudi Arabia, job satisfaction is a result of a multiplicity of intrinsic (such as appreciation, personal development, and autonomy) and extrinsic factors (such as compensation, benefits, work atmosphere, workload, and leadership behavior) [1,2]. According to a systematic review conducted in 2022 by Alotaibi et al., there is a significant variation in job satisfaction in different healthcare occupations in the Kingdom, where salary adaptability, specialty preference, and work hours are among its strongest determinants [3]. Elucidation of these complex determinants is crucial in labor planning, particularly in response to difficulties in staff turnover and quality of care in the Saudi healthcare system [4].

Not only does employee well-being depend upon job satisfaction, but employee satisfaction is a strong predictor of organizational performance, particularly in healthcare organizations. If the healthcare staff is satisfied, quality service is encouraged, turnover is reduced, and continuity of care is maximized [5,6]. Empirical research in Saudi Arabia has been able to establish that healthcare organizations where staff satisfaction is greater have higher service efficiency and patient satisfaction outcomes [4]. In addition, job satisfaction is a predictor of composite job performance, particularly in public health centers and teaching hospitals, where worker engagement has a direct effect upon outcomes in care provision [7,8].

Several studies from the Kingdom have, in fact, investigated determinants of satisfaction among healthcare providers, including allied health professionals, pharmacists, physicians, and nurses. Cited most frequently are salary, leadership behavior, workload, professional advancement, interpersonal relations, and autonomy in work. For instance, in the systematic review by Alotaibi et al., flexibility in work hours, professional advancement, and fairness in pay were cited as the most dominant determinants of satisfaction among physicians and nurses [3]. Another study from the Qassim region reported strong correlations between job satisfaction and years of experience, job rank, and daily patient load [9]. These results suggest that satisfaction among healthcare providers entails not only organizational changes in healthcare centers but also manpower strategies in healthcare occupations.

Each year, numerous workers leave their jobs due to workplace dissatisfaction [10]. The reported reasons for low job satisfaction include interpersonal relationships at work, opportunities for training and development, supervisors decision-making abilities [11], working conditions, policies, job security, and compensation [12]. Conversely, employee motivation and a supervisor’s leadership style are critical elements that significantly influence employee performance, commitment to the organization, and intention to continue in the job [13,14,15]. A study in the Qassim region reported gender, age, and workload as strong predictors of job satisfaction in healthcare providers [16]. Poor management and stress levels were reported as the main sources of discontent in Saudi healthcare [4]. Another comparative questionnaire in Jeddah reported nursing staff as being significantly less satisfied with professional support than other healthcare professionals [17].

Motivation is a process that inspires each team member to contribute their fair share, demonstrate commitment to the group, complete assigned tasks effectively, and play an effective role in the work undertaken [18]. To achieve satisfaction, each employee requires distinct motivators. Employee retention and satisfaction are not always guaranteed by employee incentives [15]. Further, when organizational leaders and institutions (including hospitals) exhibit transformational leadership behaviors—such as idealized influence, intellectual stimulation, inspirational motivation, and individualized consideration—employees report higher job satisfaction [14,19,20].

Several Saudi studies have borne testimony to how pharmacists’ satisfaction in their work has a direct reflection upon hospital functioning as well as upon patient care. For example, one study by Aldaiji et al. demonstrated how occupational stress and job satisfaction among hospital pharmacists are strongly correlated with poor quality of medication safety measures as well as greater patient risk [21]. Similarly, Almaznai et al. assessed patient satisfaction concerning pharmacy provision in Ministry of Health centers and reported that greater pharmacist work engagement was strongly associated with greater patient trust as well as better patient care outcomes in general [22]. These findings reinforce how ensuring such satisfaction in pharmacists’ work is core in guaranteeing operational efficacy as well as optimal provision of healthcare.

The pharmacy profession, particularly in Arab nations, faces similar challenges related to low job satisfaction, significantly influenced by motivation factors and leadership approaches that lead pharmacists to consider leaving their jobs [21,22]. A study conducted in Australia investigated the reasons why pharmacists leave the profession, finding that dissatisfaction with the professional environment was the primary reason, followed by a lack of career paths and opportunities, underutilization of pharmacists’ knowledge and skills, desire for change, and a disconnect from the pharmaceutical field [23]. Concerning job satisfaction among pharmacists in Saudi Arabia, a 2015 survey indicated that pharmacists, particularly those working in community pharmacies, dispensaries, and chain pharmacies, reported low levels of job satisfaction [24]. This finding contradicts research from 2005 targeting the same group (community pharmacists) [25]. These two studies examined job satisfaction among pharmacists in Saudi Arabia, with most participants desiring to leave their current positions in community pharmacy settings. Another study conducted in 2019 among pharmacists in various settings found a high satisfaction rate; it also simultaneously indicated a high intention to leave their jobs [26]. This study focused solely on pharmacists in Riyadh city.

Despite growing awareness of pharmacist job satisfaction as a significant factor, thorough research on multifaceted pharmacy work environments in Saudi Arabia has been limited. The majority of previous research has concentrated on a few regions or narrow groups, for instance, community-practicing pharmacists in Riyadh [25] or hospital pharmacists in Jeddah [27]. This limits generalizability and highlights a clear need for broader national-level investigations covering practitioners from different sectors of healthcare in order to have a clearer picture of satisfaction patterns and corresponding workforce outcomes.

Investigating job satisfaction among pharmacists and highlighting factors that influence it will help policymakers to understand the issue and take the appropriate actions to fix it. In order to capture this in our country, our approach was to reach to all pharmacists registered in Saudi Arabia. Unlike studies conducted on specific regions or certain group of pharmacists that led to conflicting results, an online survey platform developed by the Saudi Commission for Health Specialties, an independent nonprofit organization responsible for classifying and registering healthcare practitioners in Saudi Arabia including pharmacists, was utilized to assess job satisfaction from pharmacists’ perspective using the designed questionnaire.

Given the inconsistent findings related to pharmacist satisfaction in our population, which might be attributed to the selection of pharmacists in specific positions and locations, and its potential implications on healthcare system including the quality of care provided to patients, we believe that it is necessary to widen the scope of subjects to all registered pharmacists in different health sectors and highlight the factors that influence their satisfaction. Our research aims to estimate the rate of job satisfaction among all registered pharmacists in Saudi Arabia through exploring their contentment toward their jobs, their desire to leave their current positions, and the factors that influence their job satisfaction. This should help policymakers to understand the issue and take the appropriate actions when needed to fix it.

## 2. Materials and Methods

A questionnaire targeting registered pharmacists in Saudi Arabia was sent from early September to late November 2024 using the Saudi Commission for Health Specialties online platform with the aim of investigating levels of job satisfaction and the desire to quit, factors affecting job satisfaction, and methods to increase motivation. The questionnaire was designed to establish correlations between job satisfaction and participants’ demographic or professional characteristics. The study was approved by the institutional review board (IRB) at King Saud University, Riyadh, Saudi Arabia (KSU-HE-23-1171). The Saudi Commission for Health Specialties, an independent nonprofit organization responsible for classifying and registering health practitioners in Saudi Arabia, launched a new online survey platform through which an IRB-approved questionnaire can be uploaded and sent to targeted active registered practitioners in their system.

A structured questionnaire was developed in English, incorporating and adapting elements from previously validated tools used in studies on healthcare job satisfaction and motivation, including the Minnesota Satisfaction Questionnaire (MSQ), the Job Descriptive Index (JDI), and the Work Extrinsic and Intrinsic Motivation Scale (WEIMS) [28,29,30]. The instrument was tailored for the Saudi context with input from pharmacy experts.

The questionnaire comprised five sections: demographic and socioeconomic characteristics, professional personal experience, current job satisfaction and desire to quit, job satisfaction and motivation factors, and levels of job satisfaction. The demographic and socioeconomic section included information on age, gender, nationality, marital status, practicing region, qualifications, current position, organization type, monthly income, professional experience, and whether the participant currently works as pharmacist. Questions regarding average working hours, daily tasks, working system, rewards, and accomplishments were included in the professional personal experience section. Respondents provided one answer from multiple choices of yes/no/maybe questions in the first two sections. The third section consisted of two key questions. One addressed whether the participants liked their job and another was related to their intention to remain in or leave their current position. Based on their response to questions, a participant would fall into one of four groups (I don’t like my job and I have the desire to quit; I like my job but I have the desire to quit OR I don’t like my job but I don’t have the desire to quit; I like my job and I don’t have the desire to quit; Not decided whether to quit or not regardless of liking my job). The fourth section consisted of two subsections: job satisfaction and motivation factors. For each job satisfaction factor, participants were asked to indicate whether a particular factor impacts their job satisfaction with “yes” or “no” responses and rank its impact from 0 (not a factor) to 10 (highest impact). A similar approach was used for motivation factors, where participants were asked to indicate whether a particular factor impacts job motivation with “yes” or “no” responses and rank its impact from 0 to 10. The final section on job satisfaction levels included specific statements related to five main components: occupational satisfaction, working environment, empowerment, financial aspects, and occupational dissatisfaction. A Likert scale of “strongly agree,” “agree,” “neutral,” “disagree,” and “strongly disagree” was used to rate levels of job satisfaction.

The questionnaire was adapted from previous similar studies that assessed job satisfaction among pharmacists in Saudi Arabia and the broader Arab region [19,21,26]. It underwent reliability and validity testing by a group of five pharmacist experts with over five years of experience from various organizations (government hospitals, private hospitals, community, industrial, and company pharmacists). Cronbach’s alpha coefficient was used to evaluate internal consistency reliability, and the content validity index was used to assess validity. Both tests achieved satisfactory values (0.8).

Based on 30,840 registered pharmacists in Saudi Arabia [17], a minimum sample size of 380 participants was calculated with a 95% confidence interval and a ±5% margin of error. To account for potential participant withdrawal or incomplete responses, an additional 20% was added, resulting in a final target sample size of approximately 460 participants.

Descriptive statistics were employed for data analysis. Frequencies and percentages were used for categorical analyses, while the chi-squared test assessed associations between job satisfaction and demographic/professional/workplace criteria, professional personal experience, factors impacting job satisfaction, and methods of motivation. Univariate binary logistic regression was used to examine whether demographic characteristics (gender, age, occupation) could influence factors impacting job satisfaction, i.e., job promotion, positive environment, and job opportunities, with being male, age 24–29 years, and working in retail or community pharmacies as the reference categories. Multinomial logistic regression was used to predict whether job satisfaction factors or motivational methods impact job satisfaction and intent to quit with “not liking the job and intent to quit” set as the reference category. The results of logistic regression were reported as odds ratios (ORs) with 95% confidence intervals (95% CIs). SPSS software version 28 (IBM Corp., Armonk, NY, USA) was used for statistical analysis. All *p*-values were two-sided and considered significant if <0.05.

## 3. Results

### 3.1. Demographic and Socioeconomic Characteristics

A total of 330 registered pharmacists completed the survey, of whom 57% were male. The majority of participants were single (49.1%); almost an equal proportion was married (46.7%), with Saudi pharmacists comprising 77.6% of the sample. Most held a Pharm.D. degree (56.1%), worked in the central region (51.8%), and were aged between 24 and 29 and 30 to 40 years (47.9% and 40.9%, respectively). Regarding professional characteristics, most of the participants were staffing pharmacists (68.5%). Most worked in retail or community pharmacies (35.8%), governmental hospital pharmacies (22.7%), private hospital pharmacies (15.5%), and pharmaceutical companies (10.9%). More than half of participants had professional experience of ≤5 years (57.3%), while 31.8% had 5 to 10 years of experience. Average monthly incomes of SAR ≤ 9000 and SAR 9000–15,000 were reported by 45.5% and 29.7%, respectively. Notably, the majority of participants were currently working as pharmacists, while only 17.3% were working in other jobs or did not specify. More details of participant characteristics are presented in Table 1.

### 3.2. Personal Professional Experience

Most participants reported working in a shift system (59.7%). In total, 45.8% and 41.5% of participants reported working for 40–48 h and more than 48 h on average per week, respectively. Performing at least four tasks per day was reported by 60.9%, with a definite (40.9%) or possible (32.4%) sense of personal accomplishment and satisfaction at work. However, the majority believed they were neither earning enough money (70%) nor being rewarded for their efforts (66.7%). Additionally, opportunities for career growth and development were lacking (61.2%), leading approximately 42.7% to consider leaving their current job within the next five years. More details about the personal professional experience of participants are provided in Table 2.

### 3.3. Current Job Satisfaction and Desire to Quit

**Figure 1:**
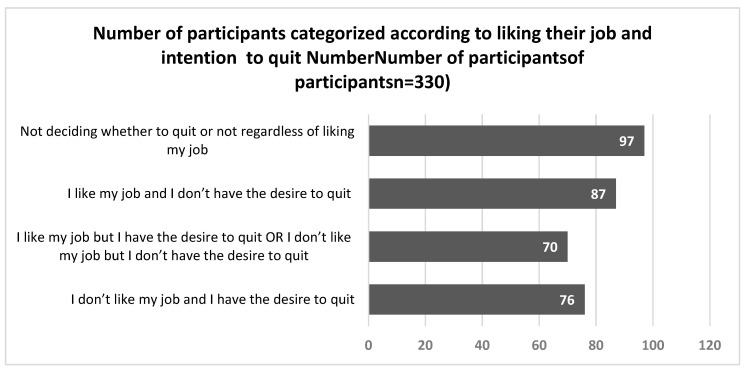
Pharmacists’ job satisfaction and intention to quit.

Two questions were used in the questionnaire to determine participants’ job satisfaction: [Do you like what you do? (Yes or No); Do you have the desire to quit your job? (Yes, No, Maybe)]. The participants were classified into four groups based on their responses, as shown in Figure 1. The responses, in order, were “undecided about quitting regardless of liking the job” (29.4%), “like their job and have no desire to quit” (26.4%), “dislike their jobs and intend to quit” (23%), and “like their job but wish to quit or dislike their job but have no desire to quit” (21.2%).

Table 3 present a classification of participants based on their responses regarding job satisfaction and intention to quit, and the impact of demographic or professional/workplace variables from their perspective. In other words, the demographic variables marital status and highest qualification significantly influenced jobs contentment and intent to quit (Table 3). With respect to professional/workplace variables, current position, organization type, monthly income, and currently working as practicing pharmacist significantly affected job satisfaction and intent to quit.

Similarly, Table 4 shows the impact of professional personal experiences on participants’ opinions towards their job and intention to quit. Job satisfaction and lack of desire to quit were significantly correlated with all components of personal professional experiences, except with the average working hours per week and the number of tasks performed per day.

More information about the impact of demographic and socioeconomic factors and participants’ professional experience on satisfaction and intention to quit are provided in Table 3 and Table 4, respectively.

### 3.4. Job Satisfaction Factors and Motivational Methods

When participants were asked to rank factors that increase job satisfaction on a scale from 0 (not a factor) to 10 (highest impact), the highest average scores were obtained for good wages (8.4/10), followed by appreciation (8.25/10), and then a positive environment (8.15/10). Regardless of scores, the factors most frequently selected by participants were a positive environment (249; 75.5%), followed by promotion (248; 75.2%) and opportunities for professional growth (200; 60.6%). Conversely, factors such as supervisor’s leadership style, employee involvement in decisions, and inspirational motivation received the lowest scores (7.82/10, 7.2/10, and 7.18/10, respectively) and were the least frequently considered as factors increasing job satisfaction. In general, factors such as good scheduling, a positive environment, supervisor’s leadership style, and job security significantly influenced participants’ responses regarding their current job satisfaction and intention to quit, as shown in Table 5.

When asked to identify methods that increase motivation using yes or no questions, most participants did not consider incentives and tuition reimbursement, coverage of transport costs and health insurance, or housing allowances as motivational. Interestingly, however, incentives and tuition reimbursement, and transport and health insurance had a significant influence on their perspective towards job satisfaction, as shown in Table 6. In other words, motivation might have minimal impact on participants’ opinions regarding job satisfaction and decisions over whether to quit from their jobs.

To further investigate the association between job satisfaction factors/motivation methods and whether participants like their job and intend to quit, multinomial logistic regression was conducted and revealed associations with good schedule (OR = 0.45, CI 95% 0.21–0.97), incentives and tuitions (OR = 3.08, CI 95% 1.45–6.57), and transport cost and health insurance (OR = 2.86, CI 95% 1.24–6.59). In other words, introducing incentives or tuition reimbursement, covering transport cost, and health insurance increased are more likely to increase the odds of job satisfaction among participants who like their job and do not want to quit compared to those who do not like their job and do intend to quit n. Interestingly, however, having a good schedule was more likely to reduce the odds of job satisfaction among participants who liked their job and did not want to quit compared to those who did not like their job and intended to quit.

**Figure 2:**
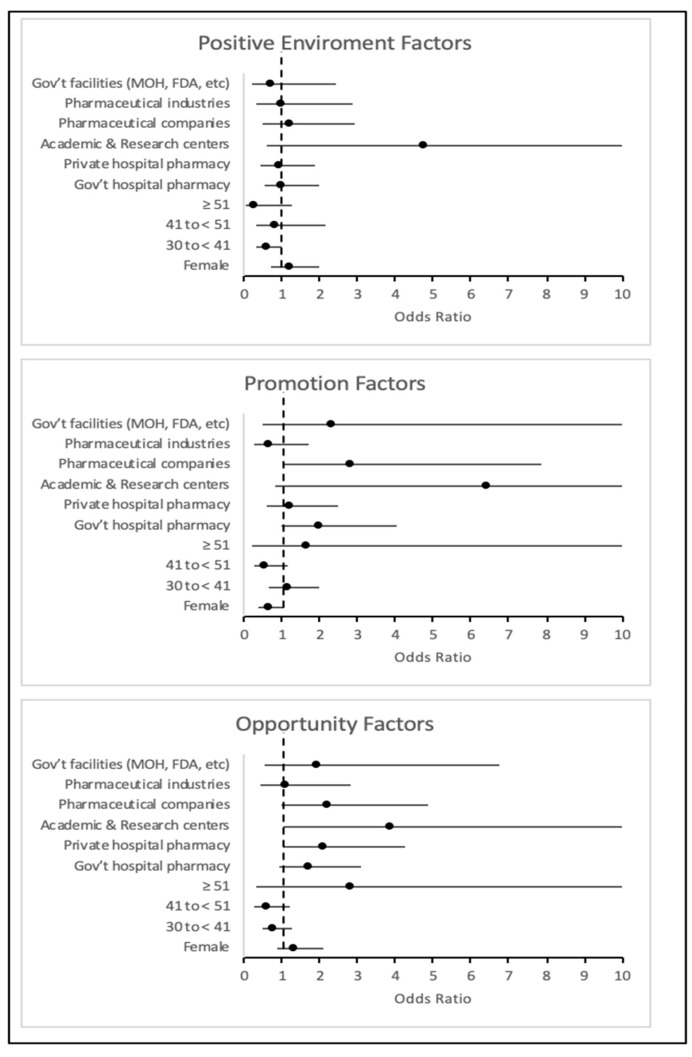
Foster plot—association between demographics and satisfaction factors.

To determine whether participants’ demographics influence their perception of promotion, a positive environment, and opportunities as factors increasing job satisfaction, a univariate regression analysis was employed to study these comparisons. With respect to promotion, being female tended to reduce the odds of considering promotion as a factor that increases job satisfaction compared to being male (OR = 0.63, CI 95% 0.383–1.045, *p* = 0.074). On the other hand, working in academia and research centers tended to increase the odds of considering promotion as a factor supporting job satisfaction compared to working in a retail or community pharmacy (OR = 6.395, CI 95% 0.810–50.462, *p* = 0.078). Meanwhile, being employed in pharmaceutical companies did significantly increase the odds of considering promotion as positive influencer of job satisfaction (OR = 2.83, CI 95% 1.020–7.866, *p* = 0.046). For the positive environment, participants aged 30 to 41 years were significantly less likely to consider a positive environment as crucial for job satisfaction compared to younger peers (OR = 0.58, CI 95% 0.338–0.994, *p* = 0.047). The same observation tended to occur with those aged ≥ 51 years (OR = 0.244, CI 95% 0.047–1.268, *p* = 0.093). Lastly, those working in private hospital pharmacies (OR = 2.12, CI 95% 1.058–4.228, *p* = 0.034) and academic/research centers (OR = 3.87, CI 95% 1.037–14.411, *p* = 0.044) significantly valued growth opportunities as a factor that increases job satisfaction compared to those working in retail or community pharmacies. The same observation was made for those working in pharmaceutical companies (OR = 2.197, CI 95% 0.991–4.868, *p* = 0.053). More details related to the results of the regression analysis are provided in Figure 2.

### 3.5. Levels of Job Satisfaction

Regarding the overall level of satisfaction with aspects of the job, participants exhibited a moderate level of occupational satisfaction, as approximately 31% to 45% responded positively to most items. Regarding the work environment, positive responses were given for all statements, with the exception of confidence in organizational leadership and workplace environment as a motivator to encourage high performance (disagreement in 32.4% and 43%, respectively). The responses to empowerment-related items were slightly more positive, with over 30% agreeing they had discretion and autonomy. With respect to the financial side from their perspective, it was the lowest, as 40% to 45% were not satisfied with what was being offered to them. Notably, occupational dissatisfaction was evident as over half of the respondents agreed or strongly agreed that staying with the organization offered few benefits. These findings suggest areas of strength in autonomy and moderate satisfaction but highlight key concerns regarding financial compensation and organizational commitment. Further details related to levels of satisfaction with different aspects of job are provided in Table 7.

## 4. Discussion

This cross-sectional study included registered pharmacists from various regions in Saudi Arabia to assess job satisfaction and the intent to quit, factors affecting job satisfaction, methods of motivation, levels of job satisfaction, and correlations between job satisfaction and participants’ demographic or professional characteristics across different workplaces. Only 26.4% reported satisfaction with their current jobs with no intention to quit. Workplace setting and monetary factors played critical roles in shaping pharmacists’ job satisfaction. Pharmacists working in public government hospitals were more likely to report lower satisfaction compared to those in pharmaceutical companies or private sectors, potentially due to rigid promotion structures, heavier workloads, and limited incentives. Similarly, lower monthly income was strongly associated with dissatisfaction and an increased intention to quit. Clearly, financial dissatisfaction is not an isolated factor but likely interacts with other workplace dynamics, particularly shift work and limited career progression, creating a stressful atmosphere that reinforces the cycle of frustration and disengagement and yields overall job dissatisfaction. Despite reporting a definite (40.9%) or possible (32.4%) personal feeling of accomplishment and satisfaction at work, only 26.4% of participants expressed satisfaction with their current jobs, while the rest were scattered, of which 23% confirmed their dissatisfaction and a clear intention to quit. A local study involving 325 pharmacists from the Riyadh region only, where 51.4% were working in governmental hospital pharmacies, revealed 62% had the intention to quit despite 39.1% reporting full job satisfaction [23]. These results align with global trends in the pharmaceutical field, where research from Australia and the Arab world has documented job dissatisfaction due to a lack of recognition, poor career structures, and unstable finances, highlighting persistent retention challenges in the Saudi pharmacy workforce [23,24,26]. Cumulatively, there is a high need for targeted reforms in compensation structures and career development frameworks in the public sector.

Job satisfaction stems from both intrinsic factors, such as achievement, recognition, and career progression, and extrinsic factors including salary, promotion opportunities, and working conditions [31]. Inadequate financial compensation was one of the most reliable indicators of discontent, as only 31.8% of participants reported being satisfied with their earnings. Similar findings were reported in previous studies. For example, Cherecheș et al., who collected and analyzed data from “online forums on Facebook and Reddit using a netnographic methodology” in 2024, found that the majority of pharmacists were dissatisfied with their salaries, believing they did not reflect their contributions [31]. Similarly, Al-Jumaili et al., who conducted a similar study among 110 pharmacists in 12 Arab countries, highlighted low salaries relative to workload as a major cause of job dissatisfaction among pharmacists [19]. A Nigerian population-based study also identified remuneration as a major factor associated with low job satisfaction, reported by 65% of pharmacists [32]. Another published study from Pakistan identified low salary and lack of incentives as major reasons for low satisfaction among pharmacists [33]. Similar findings have been reported in various studies from Saudi Arabia. For instance, Alomi et al. showed that pharmacy salaries influenced the satisfaction of pharmacists [34]. Islam and Naqvi also indicated that fringe benefits played a key role in job satisfaction among pharmacists in Saudi Arabia [35]. In addition to 70.0% of our participants believing that they were not earning enough, approximately 66.7% felt that their efforts were not rewarded. In fact, low job satisfaction and a higher intention to quit were significantly correlated with these feelings. This aligned with a previous study by Bondi et al., who reported that female pharmacists can feel discouraged if they believe their efforts are undervalued [36]. Overall, our results reiterate that good wages, rewards, recognition, and appreciation are among the most critical factors that impact motivation and increase job satisfaction.

Reaching advanced levels of education promotes greater job satisfaction. Our results showed that participants with master’s degrees, residencies, fellowships, or PhDs tended to express higher levels of satisfaction. This aligns with prior research, as higher educational attainment often correlates with better positions and autonomy, which boost satisfaction [37]. Interestingly, although Smolina et al. did not identify any relationship between education level and job satisfaction [38], our finding goes hand on hand with those reported by Aldaiji et al. in their 2020 study involving 284 Saudi pharmacists, where a positive link was found between higher education and satisfaction [21].

Considering the organization type and job position, our participants with Pharm.B. and Pharm.D. bachelor’s degrees who worked in pharmaceutical companies and industries reported significantly higher satisfaction levels compared to their counterparts working in private hospitals or community pharmacies. Ayele et al. reported that pharmacy professionals working in public hospitals had low satisfaction levels, with only 32.7% reporting job satisfaction [39]. Similarly, Aldaiji et al. reported that hospital pharmacists had lower job satisfaction than their counterparts [21]. It is well known that pharmacy professionals in demanding hospital or community roles often report heavier workloads and stress, which can reduce satisfaction [38,40]. In other words, pharmacists often experience mental strain and fatigue due to continuous workloads and insufficient support. This is confirmed by McCann et al., who identified work interruptions and heavy workloads as the main stressors in pharmacists’ jobs [41], and Lam et al., who reported that long working hours were associated with poorer satisfaction levels [42]. With respect to job position, in spite of low job satisfaction among pharmacists in administrative positions as reported by Al-Jumaili et al. [19], we found higher satisfaction levels with administrative and non-staff roles. This might be attributed to lower workloads in these positions. In other words, the nature of a work system has an influence, as approximately 60% of participants were pharmacists employed in a rotational work schedule (shift system) and were more likely to express discontent and intent to quit. This is understandable, as nonstandard, long working hours and high-pressure environments can induce fatigue and stress, significantly impacting healthcare professionals’ performance and consequently undermining their job satisfaction [38].

Organizational advancement opportunities were also pivotal for satisfaction levels. Around 61% of pharmacists reported that they were not offered sufficient opportunities for career advancement, which was strongly linked to dissatisfaction and intentions to quit. Our findings are supported by Al-Omar et al., who reported that pharmacists never expecting promotion were less satisfied compared to those expecting a promotion within a year in non-governmental pharmaceutical sectors [43]. Additionally, Butt et al. showed lack of promotion to be a major factor for dissatisfaction among pharmacists [40]. Another study by Teong W.W. et. al. revealed that a lack of promotion was a significant factor in low job satisfaction among community pharmacists [44]. While community and retail pharmacists frequently face stagnant roles with little chance for advancement, those working in academia and research and private hospital sectors are more likely to have accessible career pathways. Overall, these findings underscore the necessity for employers to implement comprehensive and open career development strategies and professional evolution to improve job satisfaction and reduce turnover among their employees.

A positive work environment, characterized by effective communication, mutual respect, and collaboration, was among the most frequently selected and highest-rated factors influencing job satisfaction. This includes good teamwork, respectful leadership, and recognition, all elements that make day-to-day work rewarding. Alzabir A. et al. reported similar findings among women employees working in public and private sectors in Sylhet City, Bangladesh, where good communication and a good relationship with supervisors were key factors for a positive work environment that increased job satisfaction [45]. Similarly, a study from Malaysia found that the work environment was the most influential factor affecting job satisfaction among pharmacists [44]. A collaborative and supportive environment increases involvement and motivation, which directly lowers the intention to quit [46]. Generally speaking, although pharmacists’ desires for advancement and ongoing development are critical for job satisfaction, the highest scores were found to be for good wages, appreciation, and a positive work environment among our participants. This indicates that while financial rewards are important, they are most effective when combined with respect, acknowledgment, and a positive work culture.

Our study contributes new knowledge by providing a national-level, sector-wide perspective from pharmacists across Saudi Arabia. Future studies should include qualitative interviews with pharmacists to better understand the root causes of dissatisfaction, as well as longitudinal follow-up to track changes over time and evaluate the impact of interventions. These efforts will help further refine policy strategies aimed at enhancing pharmacist engagement and healthcare quality.

Several limitations exist in our study; hence, our findings should be interpreted with caution. Due to its cross-sectional design, only associations and predictions can be inferred, limiting causal inference. Response bias may have been introduced by relying heavily on self-reported data, especially regarding sensitive topics such as job intentions and dissatisfaction. Although the sample was nationally representative, it was skewed toward early-career professionals, which may limit the generalizability of the findings to senior pharmacists. Additionally, achieving only an 86.8% response rate for the planned sample size calculation may further limit the generalizability of the results obtained from the included pharmacists with different perspectives. Despite these limitations, our study serves as a foundational step in a broader research initiative examining workforce satisfaction and retention trends among healthcare professionals in Saudi Arabia.

## 5. Conclusions

This study identified a notable lack of job satisfaction among Saudi pharmacists, with a significant percentage expressing discontent and plans to quit form their current positions. Financial remuneration, opportunities for career advancement, recognition, work systems, and work environments are important determinants of job satisfaction. Pharmacists with higher education, non-shift work schedules, and positions in government or pharmaceutical industries reported greater job satisfaction. Conversely, employees in private and community hospitals who worked shifts with limited promotion opportunities were more likely to report dissatisfaction. The most highly valued factors for job satisfaction include good wages, a supportive workplace, and opportunities for advancement and professional growth, emphasizing the significance of both monetary and non-monetary incentives. Such finding should be presented to healthcare related authorities, which should help in establishing a national pharmacist workforce development plan. This may include defining clear promotion criteria, offering continuing professional development (CPD) credits tied to salary bands, introducing performance-based bonuses, and benchmarking pharmacy salaries against regional and international standards. Additionally, academic institutions should align pharmacy curricula with evolving career pathways and offer early career counseling to help graduates navigate various sectors. These modifications are essential for improving job satisfaction, reducing turnover intentions, and ultimately strengthening the overall effectiveness of Saudi Arabia’s pharmacy workforce.

## Figures and Tables

**Table 1:** Demographic and professional/workplace characteristics.

Participants’ Characteristics	N (%)
Gender	
Male	189 (57.3)
Female	141 (42.7)
Age (years)	
24 to <30	158 (47.9)
30 to <41	135 (40.9)
41 to <51	31 (9.4)
≥51	6 (1.8)
Nationality	
Saudi	256 (77.6)
Non-Saudi	74 (22.4)
Marital status	
Single	162 (49.1)
Married	154 (46.7)
Divorced	13 (3.9)
Widow	1 (0.3)
Region	
Central	171 (51.8)
East	40 (12.1)
West	70 (21.1)
South	33 (10.0)
North	16 (4.8)
Highest qualification	
Unknown	7 (2.1)
Pharm.B. or undergraduate	41 (12.4)
Pharm.D.	185 (56.1)
Master	56 (17.0)
Residency/fellow	26 (7.9)
Ph.D.	15 (4.5)
Current position	
Administrative work	32 (9.7)
Senior staff [supervisor]	38 (11.5)
Staff/employee	226 (68.5)
Training unit or work	14 (4.2)
Research	14 (4.2)
Others	6 (1.8)
Organization type	
Retail or community pharmacy	118 (35.8)
Governmental hospital pharmacy	75 (22.7)
Private hospital pharmacy	51 (15.5)
Academic and research centers	15 (4.5)
Pharmaceutical companies	36 (10.9)
Pharmaceutical industries	19 (5.8)
Governmental facilities such as MOH or FDA	12 (3.6)
Others	4 (1.2)
Monthly income (SAR)	
≤9000	150 (45.5)
>9100 to 15,000	98 (29.7)
>15,000 to 21,000	43 (13)
>21,000	39 (11.8)
Professional experience (years)	
≤5	189 (57.3)
>5 to 15	105 (31.8)
>15 to 20	20 (6.1)
>20	16 (4.8)
Currently working as a practicing pharmacist	
Yes	273 (82.7)
No	57 (17.3)

**Table 2:** (a) Objective professional personal experience. (b) Subjective professional personal experience.

Participants’ Professional Experience	N (%)
(a) Objective professional personnel experience	
Average working hours per week	
<30	15 (4.5)
30–39	27 (8.2)
40–48	151 (45.8)
>48	137 (41.5)
Tasks to do in 4 h per day at work	
1 task	14 (4.2)
2 tasks	45 (13.6)
3 tasks	70 (21.2)
≥4 tasks	201 (60.9)
Work in a shift system	
Yes	197 (59.7)
No	133 (40.3)
Work as teams or as individuals at work	
Teamwork	79 (23.9)
Individual work	45 (13.6)
Both	206 (62.4)
(b) Subjective professional personnel experience	
Earning enough money	
Yes	99 (30.0)
No	231 (70.0)
Feeling rewarded for efforts	
Yes	110 (33.3)
No	220 (66.7)
Feeling of personal accomplishment and satisfaction at work	
Yes	135 (40.9)
No	88 (26.7)
Maybe	107 (32.4)
Institution provides opportunities for personal career growth and development	
Yes	128 (38.8)
No	202 (61.2)
Thinking to work for the same institution in the next 5 years	
Yes	71 (21.5)
No	141 (42.7)
Maybe	118 (35.8)

**Table 3:** (a) Correlation of job satisfaction with demographic variables. (b) Correlation of job satisfaction with professional/workplace variables.

	I Don’t Like My Job and I Have the Desire to Quit (*n* = 76)	I Like My Job but I Have the Desire to Quit OR I Don’t Like My Job but I Don’t Have the Desire to Quit (*n* = 70)	I Like My Job and I Don’t Have the Desire to Quit (*n* = 87)	Not Decided Whether to Quit or Not Regardless of Liking My Job (*n* = 97)	*p* Value
(a) Demographic variables					
Gender					0.504
Male	43 (22.8)	42 (22.2)	54 (28.6)	50 (26.5)	
Female	33 (23.4)	28 (19.9)	33 (23.4)	47 (33.3)	
Age (yrs)					0.228
24 to <30	45 (28.5)	35 (22.2)	37 (23.4)	41 (25.9)	
30 to <41	25 (18.5)	29 (21.5)	36 (26.7)	45 (33.3)	
41 to <51	4 (12.9)	4 (12.9)	13 (41.9)	10 (32.3)	
≥51	2 (33.3)	2 (33.3)	1 (16.7)	1 (16.7)	
Nationality					0.070
Saudi	66 (25.8)	56 (21.9)	61 (23.8)	73 (28.5)	
Non-Saudi	10 (13.5)	14 (18.9)	26 (35.1)	24 (32.4)	
Marital status					0.028
Single	46 (28.4)	32 (19.8)	43 (26.5)	41 (25.3)	
Married	28 (18.2)	36 (23.4)	44 (28.6)	46 (29.9)	
Divorced	2 (15.4)	2 (15.4)	0 (0.0)	9 (69.2)	
Widow	0 (0.0)	0 (0.0)	0 (0.0)	1 (100)	
Region					0.591
Central	38 (22.2)	34 (19.9)	48 (28.1)	51 (29.8)	
East	11 (27.5)	7 (17.5)	8 (20.0)	14 (35.0)	
West	18 (25.7)	15 (21.4)	19 (27.1)	18 (25.7)	
South	9 (27.3)	10 (30.0)	7 (21.2)	7 (21.2)	
North	0 (0.0)	4 (25.0)	5 (31.3)	7 (43.8)	
Highest qualification					0.035
Unknown	1 (14.3)	4 (57.1)	2 (28.6)	0 (0.0)	
Pharm.B or undergraduate	15 (36.6)	10 (24.4)	6 (14.6)	10 (24.4)	
Pharm.D.	45 (24.3)	42 (22.7)	46 (24.9)	52 (28.1)	
Master	10 (17.9)	6 (10.7)	17 (30.4)	23 (41.1)	
Residency/Fellow	3 (11.5)	4 (15.4)	12 (46.2)	7 (26.9)	
Ph.D.	2 (13.3)	4 (26.7)	4 (26.7)	5 (33.3)	
(b) Professional and workplace variables					
Current position					0.014
Administrative work	2 (6.3)	8 (25.0)	12 (37.5)	10 (31.3)	
Senior staff [supervisor]	6 (15.8)	9 (23.7)	10 (26.3)	13 (34.2)	
Staff/employee	66 (29.2)	48 (21.2)	49 (21.7)	63 (27.9)	
Training unit or work	0 (0.0)	1 (7.1)	6 (42.9)	7 (50.0)	
Research	2 (14.3)	2 (14.3)	8 (57.1)	2 (14.3)	
Others	0 (0.0)	2 (33.3)	2 (33.3)	2 (33.3)	
Organization type					<0.001
Retail or community pharmacy	0 (0.0)	1 (25.0)	3 (75.0)	0 (0.0)	
Governmental hospital pharmacy	38 (32.2)	29 (24.6)	19 (16.1)	32 (27.1)	
Private hospital pharmacy	16 (21.3)	16 (21.3)	17 (22.7)	26 (34.7)	
Academic and research centers	16 (31.4)	12 (23.5)	9 (17.6)	14 (27.5)	
Pharmaceutical companies	1 (6.7)	1 (6.7)	9 (60.0)	4 (26.7)	
Pharmaceutical industries	4 (11.1)	5 (13.9)	17 (47.2)	10 (27.8)	
Governmental facilities such as MOH and FDA	1 (5.3)	2 (10.5)	8 (42.1)	8 (42.1)	
Others	0 (0.0)	4 (33.3)	5 (41.7)	3 (25.0)	
Monthly income (SAR)					<0.001
≤9000	44 (29.3)	40 (26.7)	32 (21.3)	34 (22.7)	
>9100 to 15,000	22 (22.4)	12 (12.2)	22 (22.4)	42 (42.9)	
>15,000 to 21,000	6 (14.0)	10 (23.3)	15 (34.9)	12 (27.9)	
>21,000	4 (10.3)	8 (20.5)	18 (46.2)	9 (23.1)	
Professional Experience (years)					0.155
≤5	54 (28.6)	43 (22.8)	45 (23.8)	47 (24.9)	
>5 to 15	17 (16.2)	21 (20.0)	30 (28.6)	37 (35.2)	
>15 to 20	4 (20.0)	4 (20.0)	5 (25.0)	7 (35.0)	
>20	1 (6.3)	2 (12.5)	7 (43.8)	6 (37.5)	
Currently work as a practicing pharmacist					0.018
Yes	70 (25.6)	57 (20.9)	64 (23.4)	82 (30.0)	
No	6 (10.5)	13 (22.8)	23 (40.4)	15 (26.3)	

**Table 4:** Correlation of job satisfaction with professional experiences.

	I Don’t Like My Job and I Have the Desire to Quit (*n* = 76)	I Like My Job but I Have the Desire to Quit OR I Don’t Like My Job but I Don’t Have the Desire to Quit (*n* = 70)	I Like My Job and I Don’t Have the Desire to Quit (*n* = 87)	Not Decided Whether to Quit or Not Regardless of Liking My Job (*n* = 97)	*p* Value
Factors related to workload					
Average of working hours per week?					0.083
<30	4 (26.7)	3 (20.0)	5 (33.3)	3 (20.0)	
30–39	3 (11.1)	6 (22.2)	13 (48.1)	5 (18.5)	
40–48	33 (21.9)	28 (18.5)	45 (29.8)	45 (29.8)	
>48	36 (26.3)	33 (24.1)	24 (17.5)	44 (32.1)	
Tasks to do in 4 h per day at work					0.187
1 task	4 (28.6)	3 (21.4)	5 (35.7)	2 (14.3)	
2 tasks	9 (20.0)	12 (26.7)	10 (22.2)	14 (31.1)	
3 tasks	11 (15.7)	12 (17.1)	28 (40.0)	19 (27.1)	
≥4 tasks	52 (25.9)	43 (21.4)	44 (21.9)	62 (30.8)	
Work in a shift system					<0.001
Yes	60 (30.5)	44 (22.3)	42 (21.3)	51 (25.9)	
No	16 (12.0)	26 (19.5)	45 (33.8)	46 (34.6)	
Work as teams or as individuals at work					0.005
Teamwork	14 (17.7)	19 (24.1)	28 (35.4)	18 (22.8)	
Individual work	18 (40.0)	12 (26.7)	4 (8.9)	11 (24.4)	
Both	44 (21.4)	39 (18.9)	55 (26.7)	68 (33.0)	
Factors related to personnel compensation					
Earning enough money					<0.001
Yes	11 (11.1)	20 (20.2)	46 (46.5)	22 (22.2)	
No	65 (28.1)	50 (21.6)	41 (17.7)	75 (32.5)	
Feeling rewarded for efforts					<0.001
Yes	3 (2.7)	19 (17.3)	59 (53.6)	29 (26.4)	
No	73 (33.3)	51 (23.2)	28 (12.7)	68 (30.9)	
Feeling of personal accomplishment and satisfaction at work					<0.001
Yes	3 (2.2)	33 (24.4)	65 (48.1)	34 (25.2)	
No	53 (60.2)	17 (19.3)	4 (4.5)	14 (15.9)	
Maybe	20 (18.7)	20 (18.7)	18 (16.8)	49 (45.8)	
Factors related to career development					
Institution provides opportunities for personal career growth and development					<0.001
Yes	12 (9.4)	22 (17.2)	61 (47.7)	33 (25.8)	
No	64 (31.7)	48 (23.8)	26 (12.9)	64 (31.7)	
Thinking about working for the same institution in the next 5 years					<0.001
Yes	6 (8.5)	12 (16.9)	42 (59.2)	11 (15.5)	
No	61 (43.3)	37 (26.2)	8 (5.7)	35 (24.8)	
Maybe	9 (7.6)	21 (17.8)	37 (31.4)	51 (43.2)	

**Table 5:** Job satisfaction factors.

	All Participants (n = 330)	I Don’t Like My Job and I Have the Desire to Quit (*n* = 76)	I Like My Job but I Have the Desire to Quit OR I Don’t Like My Job but I Don’t Have the Desire to Quit (*n* = 70)	I Like My Job and I Don’t Have the Desire to Quit (*n* = 87)	Not Deciding Whether to Quit or Not Regardless of Liking My Job (*n* = 97)	*p* Value
Factors increase job satisfaction						
Promotion						0.245
Yes	248 (75.2)	62 (25.0)	47 (19.7)	65 (26.2)	74 (29.8)	
No	82 (24.8)	14 (17.1)	23 (28.0)	22 (26.8)	23 (28.0)	
Good schedule						0.002
Yes	186 (56.4)	56 (30.1)	38 (20.4)	49 (26.3)	43 (23.1)	
No	144 (43.6)	20 (13.9)	32 (22.2)	38 (26.4)	54 (37.5)	
Good wage						0.579
Yes	176 (53.3)	45 (25.6)	35 (19.9)	43 (24.4)	53 (30.1)	
No	154 (46.7)	31 (20.1)	35 (22.7)	44 (28.6)	44 (28.6)	
Good teamwork						0.264
Yes	194 (58.8)	45 (23.2)	36 (18.6)	58 (29.9)	55 (28.4)	
No	136 (41.2)	31 (22.8)	34 (25.0)	29 (21.3)	42 (30.9)	
Positive environment						0.038
Yes	249 (75.5)	64 (25.7)	47 (18.9)	70 (28.1)	68 (27.3)	
No	81 (24.5)	12 (14.8)	23 (28.4)	17 (21.0)	29 (35.8)	
Opportunities						0.310
Yes	200 (60.6)	53 (26.5)	41 (20.5)	49 (24.5)	57 (28.5)	
No	130 (39.4)	23 (17.7)	29 (22.3)	38 (29.2)	40 (30.8)	
Appreciation						0.164
Yes	182 (55.2)	47 (25.8)	31 (17.0)	48 (26.4)	56 (30.8)	
No	147 (44.5)	29 (19.7)	39 (26.5)	39 (26.5)	40 (27.2)	
A supervisor’s leadership style						0.048
Yes	153 (46.4)	42 (27.5)	35 (22.9)	42 (27.5)	34 (22.2)	
No	177 (53.6)	34 (19.2)	35 (19.8)	45 (25.4)	63 (35.6)	
Inspirational motivation						0.062
Yes	110 (33.3)	35 (31.8)	22 (20.0)	25 (22.7)	28 (25.5)	
No	220 (66.7)	41 (18.6)	48 (21.8)	62 (28.2)	69 (31.4)	
Job security						0.007
Yes	159 (48.2)	42 (26.4)	29 (18.2)	52 (32.7)	36 (22.6)	
No	171 (51.8)	34 (19.9)	41 (24.0)	35 (20.5)	61 (35.7)	
Involve employees in decisions						0.059
Yes	110 (33.3)	35 (31.8)	19 (17.3)	27 (24.5)	29 (26.4)	
No	220 (66.7)	41 (18.6)	51 (23.2)	60 (27.3)	68 (30.9)	

**Table 6:** Job motivation methods.

	All Participants (*n* = 330)	I Don’t Like My Job and I Have the Desire to Quit (*n* = 76)	I Like My Job but I Have the Desire to Quit OR I don’t Like My Job but I Don’t Have the Desire to Quit (*n* = 70)	I Like My Job and I Don’t Have the Desire to Quit (*n* = 87)	Not Decided Whether to Quit or Not Regardless of Liking My Job (*n* = 97)	*p* Value
Methods to increase motivation						
Incentives and tuition						
Yes	127 (38.5)	20 (15.7)	25 (19.7)	46 (36.2)	36 (28.3)	
No	203 (61.5)	56 (27.6)	45 (22.2)	41 (20.2)	61 (30.0)	0.005
Transport costs and health insurance						
Yes	110 (33.3)	17 (15.5)	23 (20.9)	38 (34.5)	32 (29.1)	
No	220 (66.7)	59 (26.8)	47 (21.4)	49 (22.3)	65 (29.5)	0.04
Allowance for a home						
Yes	86 (26.1)	16 (18.6)	19 (22.1)	22 (25.6)	29 (33.7)	
No	192 (58.2)	60 (24.6)	51 (20.9)	65 (26.6)	68 (27.9)	0.615

**Table 7:** Job satisfaction levels.

Statement	Strongly Disagree, *n* (%)	Disagree, *n* (%)	Neutral, *n* (%)	Agree, *n* (%)	Strongly Agree, *n* (%)
Occupational satisfaction					
I enjoy going to work every day	45 (13.6)	49 (14.8)	134 (40.6)	70 (21.2)	32 (9.7)
My job gives me a sense of accomplishment	42 (12.7)	66 (20.0)	81 (24.5)	96 (29.1)	45 (13.6)
I’m content with my work	47 (14.2)	51 (15.5)	84 (25.5)	99 (30.0)	49 (14.8)
Others would consider my job a fortunate opportunity	52 (15.8)	48 (14.5)	108 (32.7)	77 (23.3)	45 (13.6)
If I could start over, I would choose this job again	74 (22.4)	46 (13.9)	73 (22.1)	81 (24.5)	56 (17.0)
Working environment					
I have confidence in the leadership of this organization	63 (19.1)	44 (13.3)	106 (32.1)	72 (21.8)	45 (13.6)
To contribute to the success of my workplace, I am prepared to put in more effort than is often required	42 (12.7)	34 (10.3)	77 (23.3)	100 (30.3)	77 (23.3)
My work environment motivates me to provide my best job performance	75 (22.7)	67 (20.3)	92 (27.9)	65 (19.7)	31 (9.4)
Overall, I am satisfied with my work environment	52 (15.8)	54 (16.4)	110 (33.3)	80 (24.2)	34 (10.3)
Empowerment					
I have enough discretion in my job to rely on my judgment	36 (10.6)	46 (13.9)	114 (34.5)	104 (31.5)	31 (9.4)
I have the freedom to choose any working approach to do the job	49 (14.8)	73 (22.1)	89 (27.0)	93 (28.2)	26 (7.9)
Financial aspect					
I’m content with my pay	87 (26.4)	64 (19.4)	74 (22.4)	73 (22.1)	32 (9.7)
I am pleased with the additional benefits provided by my job	75 (22.7)	57 (17.3)	88 (26.7)	67 (20.3)	43 (13.0)
Occupational dissatisfaction					
Staying with this organization doesn’t offer much in the way of benefits	37 (11.2)	37 (11.2)	87 (26.4)	86 (26.1)	83 (25.2)
Deciding to work for this institution was a grave error	75 (22.7)	83 (25.2)	95 (28.8)	48 (14.5)	29 (8.8)

## Data Availability

The data presented in this study are available on request from the corresponding author due to privacy and legal requirements.

## Abbreviations

The following abbreviations are used in this manuscript:

SAR	Saudi Riyals (Saudi Currency)
Pharm.B.	Bachelor of Pharmacy
Pharm.D.	Doctor of Pharmacy
MOH	Ministry of Health
FDA	Food and Drug Administration
